# Supplementary material for: Improved dielectric performance of graphene oxide reinforced plasticized starch
Source: PLoS One. 2024 Dec 30;19(12):e0309469. doi: 10.1371/journal.pone.0309469 (PMC11684599; doi:10.1371/journal.pone.0309469)
Supplement: Attachment — Submitted filename: PLOS-Reviewers _ response_R1.docx [file pone.0309469.s001.docx]

**Reply to the Referees Comments on Manuscript Number: PONE-D-24-13185**

We thank the referees for the critical review of the manuscript. In the following, we provide our responses to the referee’s comments.

**Reply to the First referee’s comments:**

**Comment (1)**   You need to add some references in the introduction part:
1-https://doi.org/10.1016/j.carpta.2022.100239
2-https://doi.org/10.3390/polym16020294
3-DOI:10.1007/s13762-020-02764-3
4- DOI: 10.3390/gels9050416

**Response 1:** We thank the referee for the comment. Following the referee’s suggestion the relevant articles have been included in the revised version of the manuscript. Please see the list of changes #1.

**Reply to the Second referee comments:**

**Comment (1)** Manuscript Title: Improved Dielectric Performance of Graphene Oxide reinforced Starch biopolymer.

The authors used from the first line of the abstract the expression ‘plasticized starch’ so it would be better to replace starch biopolymer to plasticized starch to be:
“Improved Dielectric Performance of Graphene Oxide Reinforced to Plasticized Starch”
And all the initials are in capital letters.
There is a great difference between starch and plasticized starch.

**Response 1:** We thank the referee for the comment. We follow the instructions and write the title accordingly. Please see the list of changes # 2.

**Comment (2)**   Abstract: The authors started the abstract directly to the methods used and the materials. The abstract should give first a brief background about the idea/problem this work would help to solve it, the methods, results and finally the conclusion.

**Response 2:** We thank the referee for the comment. A brief background about the idea/problem of this work has been added in the revised version of the manuscript. Please see the list of changes # 3.

**Comment (3)**     Keywords: The word starch should be changed to plasticized starch.

**Response 3:** We thank the referee for the comment. Following referee’s comments the plasticized starch has been included. Please see the list of changes # 4.

**Comment (4)** Novelty: The authors should emphasis the novelty of their work as there are many research articles with similar ideas and the same measurements. Moreover, they should clearly compare their works in this manuscript with that already published using GO/PS and show the advantage of GO over the reduced GO in their work and applications.

**Response 4:** We thank the referee for the valuable comment. To the best of our knowledge, the dielectric performance of PS/GO is still to be done. Therefore, this work is fully intended to investigate the complete dielectric properties where the influence of GO on the PS matrix is keenly focused. Please see the list of changes # 5.

**Comment (5)**   Introduction: A little confusing and needs to be reorganized. Some abbreviations are written directly in the text without mentioning the name first. In addition, their work is focusing on the improvement of the dielectric performance of the prepared GO/PS, there is a poor information about important of this issue in the introduction

**Response 5:** We thank the referee for the comment. For better understanding and clarity, the text in the introduction in the manuscript was thoroughly revised, and reorganized with proper elaboration of abbreviations. Please see the list of changes #6

**Comment (6)** Materials and methods: Need references in each step/paragraph. In section 2.3: ac conductivity [use capital letters; AC].
There isn’t any clear sentence that give the number of replications in each measurement or standard deviation.

**Response 6:** We thank the referee for the comment. Following the suggestion corrections has been made in the revised manuscript. Please see the list of changes #7

**Comment (7)** Results and discussion: Figures captions are very short. They need to be more descriptive. The discussion should contain some of the previous works that agree and also that disagree of the author’s work.
The FTIR spectra in Figure. 2 are noisy/ so thick. Moreover, a table that contains the band’s center and the corresponding band assignment should be given. This section wasn’t clear enough in the text.

**Response 7:** We thank the referee for the comment. A table of FTIR data has been added and changes in the text have been made. Please see the list of changes #8

**Comment (8)** English language of the manuscript needs to be revised and corrected, there are many grammar mistakes, examples in the introduction section:
building a energy, a issue

**Response 8:** We thank the referee for the comment. The grammatical mistakes, spelling mistakes, and all references were checked thoroughly and formatted properly. Please see the list of changes #9.

**Comment (9)** The references should be updated, some of them are very old.

**Response 9:** We thank the referee for the comment. Following suggestion several relevant articles have been cited in the revised manuscript. Please see the list of changes #10

**List of Changes on Manuscript ID PONE-D-24-13185**

1. The references 3 has been added and 5, and 7 has been changed to the revised manuscript.
2. The Manuscript Title has been changed where all the initials are in capital letters in the revised manuscript.
3. A brief background about the idea/problem of this work has been added in the revised version of the manuscript as “High dielectric constant with less dielectric loss composites are highly demandable for technological advancements across various fields, including energy storage, sensing, and telecommunications. Their significance lies in their ability to enhance the performance and efficiency of a wide range of devices and systems.”
4. The word starch in “keywords” has been changed to plasticized starch.
5. Novelty and comparison with other works are already mentioned in the introduction section of the revised manuscript as “Reported work on starch-based materials so far: improvement of mechanical, electrochemical, optical, and thermal properties [27-29]. To the best of our knowledge, the dielectric performance of PS/GO is still to be done. Therefore, this work is fully intended to investigate the complete dielectric properties where the influence of GO on PS matrix is keenly focused.” Reference 33 is added which was published on dielectric performance of plasticized starch/reduced graphene oxide (PS/rGO) nanocomposite.
6. For better understanding and clarity, the text in the introduction in the manuscript was thoroughly revised, reorganized with proper elaboration of abbreviation. For the better background of importance of dielectric performance of the prepared GO/PS, there is a completely new paragraph has been added in the introduction “The electronics and energy storage industries are progressing where electronic circuits have to adapt by becoming smaller, more integrated, light weight and smarter to keep pace with globalization trends alongside green electronics. Here biopolymer as dielectric materials are leading as increased interest not because they offer advantages such as low dielectric loss, high breakdown strength but also it has practical advantage over heavy metal, conventional silicon di-oxide based device, ceramics or synthesized polymer along with easy processing and diverse structures, low weight. Among various industrial applications, they have the advancements as insulator particularly used in organic field effect transistor (OFET), wind turbine, aerospace, memory storage, energy storage, wastewater remediation and so on. However, biopolymer must have high dielectric constant having the withstand capability with high electric field, and thermo-mechanical stability along with high dielectric constant and less dielectric loss. Particularly in energy storge device, as energy is directly proportional to the dielectric constant and the electric field, building energy stored device with high dielectric constant can provide more energy to store. A single biopolymer has low dielectric constant with poor thermal stability which has limited its application. These are the reasons that there has been a surge in research aimed at developing polymer-based dielectric composites with superior properties, including high dielectric constant, low dielectric loss, and high breakdown strength simultaneously. These aforementioned advantages are warranted in organic layer polymer.”
7. All ac is changed with AC and reference 30 is added.
8. We elaborate the figures captions. As there is no research in dielectric performance of PS/GO, therefore no comparison was possible. We have added a FTIR data containing table in Table 1.
9. The grammatical mistakes have been corrected.
10. The references 1, 3, 5, 7, 32, and 33 have been added to the revised manuscript.
