# Supplementary material for: Improved dielectric performance of graphene oxide reinforced plasticized starch
Source: PLoS One. 2024 Dec 30;19(12):e0309469. doi: 10.1371/journal.pone.0309469 (PMC11684599; doi:10.1371/journal.pone.0309469)
Supplement: Attachment — Submitted filename: PONE-D-24-13185 revised.docx [file pone.0309469.s002.docx]

**Reviewer Comments** for the revised manuscript number: **Manuscript Number:** PONE-D-24-13185

“Improved Dielectric Performance of Graphene Oxide Reinforced Plasticized Starch”

Submitted to Journal: PLOS ONE Journal

Dear Editor,

The manuscript now is acceptable. **All required questions have been answered and all responses meet formatting specifications.**

Thank you.

Kind Regards

Gehan A-R Ahmed

Prof. Molecular Biophysics

Spectroscopy Department

Physics Research Institute

National Research Centre

Cairo- Egypt
