# Supplementary material for: Improved dielectric performance of graphene oxide reinforced plasticized starch
Source: PLoS One. 2024 Dec 30;19(12):e0309469. doi: 10.1371/journal.pone.0309469 (PMC11684599; doi:10.1371/journal.pone.0309469)
Supplement: Attachment — Submitted filename: PLOS-Reviewers _ response_R2.docx [file pone.0309469.s003.docx]

**Reply to the Referees Comments on Manuscript Number: PONE-D-24-13185R1**

We thank the referees for the critical review of the manuscript. In the following, we provide our responses to the referee’s comments.

**Reply to the Second referee comments:**

**Comment (1)** the 1st line in abstract, are should be is.

**Response 1:** We thank the referee for the comment. We fixed the error in the abstract. Please see the list of changes # 1.

**Comment (2)**   The statement “in this work……: graphene oxides to graphene oxide,, different concentration to different concentrations,, and were studied to was studied. XRD section: The diffraction peaks at……., confirms should be confirm, and this works should be this work, and so on.

**Response 2:** We thank the referee for the comment. We fixed the errors in the revised version of the manuscript. Please see the list of changes # 2.

**Comment (3)**     Figure 4 represents the effect of GO nanofiller on the changes of dielectric constant, ε of PS. ε for Pure PS. ε is almost similar for PS to low and high frequency.( this sentence needs to be revised, This sentence is incomprehensible

**Response 3:** We thank the referee for the comment. We changed the line in the revised version of the manuscript. Please see the list of changes # 3.

**Comment (4)** The manuscript needs careful linguistic review, taking into account the plural, singular, and appropriate tenses.

**Response 4:** We thank the referee for the valuable comment. The grammatical mistakes, spelling mistakes, and all references were checked thoroughly and formatted properly. Please see the list of changes #4.

**List of Changes on Manuscript ID PONE-D-24-13185R1**

1. In the abstract, the line “High dielectric constant with less dielectric loss composites are highly demandable for technological advancements across various fields, including energy storage, sensing, and telecommunications.” has changed to “*High dielectric constant with less dielectric loss composites is highly demandable for technological advancements across various fields, including energy storage, sensing, and telecommunications*”.
2. In the abstract the line “In this work, the dielectric performance of graphene oxides (GO) reinforced plasticized starch (PS) nanocomposites (PS/GO) for different concentrations of GO nanofiller were studied.” has changed to “*In this work, the dielectric performance of graphene oxide (GO) reinforced plasticized starch (PS) nanocomposites (PS/GO) for different concentrations of GO nanofiller were studied.*”.
3. In section 3.4.1 of the revised manuscript, the lines “Figure 4 represents the effect of GO nanofiller on the changes of dielectric constant, *ε* of PS. ε for Pure PS. *ε* is almost similar for PS to low and high frequency.” has changed to “*Figure 4 represents the effect of GO nanofiller on the dielectric constant (ε) of PS. For PS, the value of ε does not show a large variation as the frequency changes.*
4. The grammatical mistakes have been corrected.
